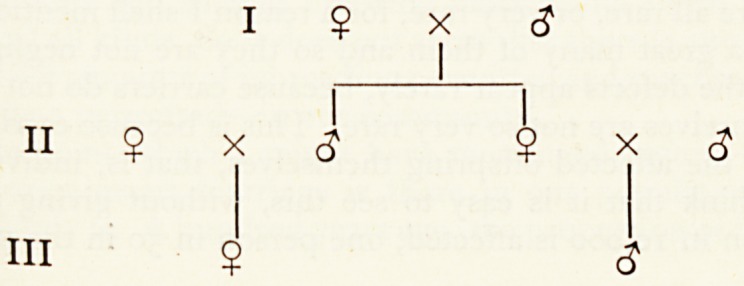# Cousin Marriage

**Published:** 1955-09

**Authors:** J. A. Fraser Roberts

**Affiliations:** Director, Burden Mental Research Department, Stoke Park Colony, Bristol


### COUSIN MARRIAGE

FORTY-SECOND LONG FOX MEMORIAL LECTURE DELIVERED ON 20TH OCTOBER, 1953

BY

J. A. FRASER ROBERTS, M.A., M.D., D.SC., F.R.C.P.
*Director, Burden Mental Research Department, Stoke Park Colony, Bristol*

It is a great honour to be invited to contribute a lecture to this long-established series, founded to commemorate a very eminent physician of the Bristol Medical School and a member of a family distinguished for services to medicine and to the City over many years. I have also seen with particular pleasure that the first Long Fox lecture was given by John Beddoe, a pioneer of British anthropology and one of the greatest men of science that this City has produced. His writings, particularly *The Races of Britain*, have been an inspiration; and, like many others, I have turned to his tables as a source of material. I have been grateful, too, to have had available at the University library the Beddoe collection of books, which contains some rare and useful works.

Turning to the subject of this lecture—cousin marriage—you will naturally expect a biologist to deal with the biological implications and consequences of the marriage of blood relatives; and this indeed is what I propose to do. But before starting on the main subject I should like to spend some of the time on more general questions; for in addition to biological implications there are others—for example, the sociological—and these other aspects of our subject are important and, I think, interesting.

There can be few if any stronger taboos than those prohibiting the marriage of certain relatives. The scope of the prohibited degrees has varied widely at different times and in different places, sometimes extending to relationships which seem remote to us. The closest relationships, however, for example brother and sister, or parent and child, have at almost all times and in almost all places been considered an absolute bar. The very few exceptions only serve to emphasize the universality of something deeply rooted in the mind of man from very early times. The exceptions in fact attract undue notoriety by their very rarity. The brother-sister marriages amongst some of the Pharaohs arose from dynastic considerations, for it was held that a wife of a royal line transmitted the right of kingship to her children, and princes had an unquestionable right to the throne only if they were born of a father and mother who themselves had equal rights. It has been said that during the times of the Ptolemys the practice spread through the population, but such instances only emphasize how fleeting have been the departures from what many would no doubt hold to be a part of natural law.

Is there any stronger taboo than that against incest? It is difficult to think of one. Are there any other laws so relatively easily broken and yet so universally respected? But how have the taboos come to be established? What purpose do they serve? There are two explanations, the genetic and the sociological, or rather, I should say, the psychological-sociological. The genetic explanation, which seems very unlikely, is that from very early times mankind became aware of evil consequences arising from inbreeding, both in his own species and in his domestic animals. The sociological explanation is that the prohibitions are calculated to preserve the stability of the family. In any society the family is the all-important unit, and nothing could be more destructive of its harmony than the possibility of unions between its members. This is what Malinowski* says: "The main sociological reason for marriage taboos and prohibited degrees is the elimination of sex from relations of the family type. A group

* For this and a number of other references, I am indebted to " Kindred and Affinity as Impediments to Marriage, being the report of a commission appointed by His Grace the Archbishop of Canterbury ". London, S.P.C.K., 1940.

leading a joint life with the intimacy of daily concerns, with the need of an organized authority and unselfish devotion, cannot tolerate within its framework the possibilities of sexual approaches, for these act as a competitive and disruptive force incompatible with the even tenor and stability of the family."

It is easy to see how well the psychological-sociological explanation fits the facts and how badly the genetic. The extension of prohibitions from the universally agreed close relationships to those which are distant can be seen to follow closely the pattern of family life—of what at a particular time and in a particular place constitutes the family group. For example, in some matrilineal societies marriages to aunts or cousins on the mother's side are strictly forbidden, whereas genetically equivalent marriages to a cousin on the father's side or to the father's sister are freely allowed, and may indeed be considered positively desirable. Or, to take another example, first cousins, the children of brothers and sisters, have often been considered as the equivalent of brothers and sisters. When this has been so, marriage between first cousins has been as much barred as marriage between brothers and sisters.

Then again, the genetic explanation can only apply to blood-relationship and not at all to relationship by affinity, that is, relationship created by marriage. There can be no genetic reason why a man should not marry his son's wife, for they have no biological connexion, yet the prohibitions apply just as strongly to affinity as to blood-relationship.

If we turn to what is allowed and what is not allowed here and now, we see at once that it does not make genetic sense, though it does make sound sociological sense. In this country a man may not marry his niece; but to anticipate what I shall be talking about a little later, he can do what is exactly equivalent genetically, namely, marry his double first cousin (double first cousins arise when, say, two brothers marry two sisters). Of course, a man may not marry his sister, yet it is possible for him, genetically speaking, to do just that, and perfectly legally. Suppose a pair of identical twin brothers were to marry a pair of identical twin sisters—not a far-fetched possibility. Speaking genetically, the offspring of the two couples are the same as brothers and sisters. Yet there is no legal impediment to the son of one couple marrying the daughter of the other.

The idea that mankind, from primitive man onwards, observed that bad results came of inbreeding, either in his own species, or in his domesticated animals, so giving rise to the taboos, is somewhat fantastic. First of all, it is doubtful how far he would have observed it; he certainly could not have observed it as a constant phenomenon. Some of the Pharaohs who came of quite a long line of marriages of very close relatives, were persons of the highest qualities, both physically and mentally. Thus, at the beginning of the Eighteenth Dynasty, perhaps the most brilliant in the long history of Egypt, there was a succession of highly gifted, and also highly inbred, rulers. Queen Hatshepsut, outstanding even in that company, was the daughter of half brother and sister and her mother Aahmes was the result of two successive marriages of full brothers and sisters. Of course, I am not suggesting for a moment that such marriages might in any circumstances be a good thing even from the narrow genetic point of view; all I am doing is emphasizing how thin is the attempt to explain the origin of the prohibitions on genetic lines.

It is true that in ancient writings statements can be found to the effect that the offspring of unions of blood-relatives tend to turn out badly; but on the whole I imagine that such references are rather rare. Pope Gregory the Great in a letter to St. Augustine refers to heathen laws permitting marriages between brothers and sisters and first cousins. He says: "We have learned from experience that the children of such marriages do not do well." Benedict the Levite, writing in the year 842, says: "Such marriages are the cause of physical defects in the children"—an interesting observation. The impression one gets, however, is that the writers were very naturally finding additional reasons to support prohibitions already established on other grounds. And, of course, there is a reason, though not an intrinsic one, why in man poor results might sometimes be observed. People who defy such universally respected rules must often be peculiar. They may transmit undesirable qualities to their offspring by way of inheritance, and are also likely to provide them with a poor home in which to grow up.

I am tempted here to digress a little and say a word about biological superstitions. There are those who say that ancient folklore and widely held popular beliefs are very likely always to have something in them, and that they should be treated with great respect. This was the view of an old friend of many years ago, who was a professor of chemistry. Now it is rather easy to be tolerant of superstitions in someone else's science; a professor of chemistry is unlikely to encounter a popular superstition to the effect that common salt is not composed of sodium and chlorine. At the present time the genetic theories of Lysenko seem to be most wholeheartedly supported by those least hampered by a knowledge of the subject. There are in fact biological superstitions which go back thousands of years and which are widespread even today, but which, nevertheless, are devoid of any foundation. A much more widespread popular belief than the evil consequences of inbreeding, which has something in it, is one which has nothing in it—namely, maternal impressions, the belief that the unborn child may be affected in some corresponding way by strong impressions made upon the mother. Of course, there is a case for investigating popular beliefs and Darwin investigated this one. He got a friend at a large maternity hospital to question some hundreds of women, before the birth of the child, as to strong impressions during their pregnancy. In not one single instance were any of these reflected in anything wrong with the child, or anything outstanding about the child. But, of course, after the child was born the mothers thought of all kinds of associations. Professor Darlington, in a recently published book*, gives an example showing how widespread this erroneous belief still is, even amongst educated people. Some years ago a lady wrote to the Tate Gallery, asking whether she could have a good look at Millais's well-known picture, " Sir Isumbras at the Ford ". At the time, for some reason, it was stored away. She was sure that if she could contemplate the picture her unborn son would look like the two delightful children, whom, you may remember, the artist also included. Well, biology is a funny science, and as things turned out, it wasn't a son—it was a daughter —and what she looked like was the horse. The much stronger belief has in fact led to little if anything in the way of rules of conduct; the lesser one, a belief in the evil effects of inbreeding, cannot be responsible for so important and mandatory a set of social rules.

So we are left with customs and laws which, we may agree, have a sound and useful sociological basis. The present laws of this country seem well adjusted to their purpose—the preservation of family life, without undue or unreasonable individual hardship. Only relatively close blood-relationship is a bar, or close affinity created by marriage. Of course, during a period of change there are liable to be difficulties. For some centuries during which the family unit was becoming smaller and progressively more restricted to closer relatives, the law tended to lag behind and the more widely drawn prohibitions of earlier times led to much difficulty. But it all seems to have settled down now. The Roman Catholic Church has attained the same end in a somewhat different way. They regard certain very close relationships as constituting a universal and unalterable bar. Others are dispensable. Even a brother-sister marriage is theoretically dispensable, but, say the canonists, no dispensation ever has been given for such a marriage, and none ever will be. Dispensations for the marriage of uncle and niece, in countries where such a marriage is legal, are much discouraged, are granted very rarely, and only for a good reason. Dispensations are freely given, I believe, for the marriage of first cousins.

There is little if anything left in the way of social problems, but there are biological consequences and implications and it is to these that I want to devote the rest of this lecture. As a preface I should like to say this: I should not like anyone to get the impression that I subscribe for a moment to the belief that we are wiser or cleverer than our predecessors. There is nothing more ridiculous than the dwarf on the giant's shoulder saying " Look how much taller I am than papa ". Rather, what most of us,

* *The Facts of Life*. London, Allen and Unwin. 1953.

I am sure, often feel is: "How I wish Newton, or Harvey, or Galton, had known about this; I wonder what he would have made of it." Science progresses by building up on foundations already laid; but, to change the metaphor, there come moments of illumination, and then nothing can ever be the same again. A key is discovered and a door is opened to a whole new world. In biology such a transcendent moment came in 1900 with the rediscovery of the work of Gregor Mendel. To quote Professor Darlington once again: " Before the year 1900, we may say, almost all that we believed of the principles of heredity that was not in Lucretius was wrong." The one exception was Mendel, and he was before his time. Darwin was not; in fact in regard to heredity he saw no further than the rest. So it is in the light of the transformation of 1900 and what has been learned since that we must assess the biological implications of the marriage of blood-relatives.

May I remind you of a very few basic principles of genetics which must underlie our discussion. The body is made up of cells. Each cell contains a nucleus, which is its directing mechanism. Within each human nucleus lie twenty-four pairs of chromosomes, all of characteristic size and shape. Along the length of the chromosome filaments are arranged the genes. They are in linear order: as is often said, like beads on a string. The total number is very large—many thousands, or indeed, more probably, many tens of thousands. The genes are the hereditary determinants. Everything that the parent contributes to the child by way of organic inheritance is contained in the gametes, the ovum and the spermatozoon, which are single cells; and the whole of that contribution is represented by the complement of genes. So if we follow the very simple essentials of chromosome behaviour, we have the key to understanding heredity.

When, during development, an ordinary cell division takes place, the forty-eight chromosomes, and the genes they contain, have doubled. Each filament has laid down alongside itself an exact copy. When the mother cell divides, giving two daughter cells, the doubled filaments separate, and so the daughter cells contain in their nuclei the very same chromosomes and borne upon their length the very same complement of genes.

Prior to the formation of the gametes, however, a division of a special type, the reduction division, takes place. The consequence is that the members of each pair of chromosomes separate bodily, so that an ovum or spermatozoon contains, not forty-eight chromosomes, but twenty-four, one derived from each pair. When fertilization takes place, each chromosome from the sperm nucleus pairs up with the corresponding chromosome from the nucleus of the ovum, and so there is reconstituted in the new individual the full somatic number of twenty-four pairs, one member of each pair having been contributed by the father and one by the mother. Starting with the all-important fact that the genes are paired, we see that if we think of one particular gene, on one particular chromosome of one particular pair, the chance that the mother—or the father—will contribute that particular gene to a particular child is one-half. We can extend this to all the genes, so parent and child have half their genes in common.

The same thing is true of brothers and sisters. Let us call the alternative genes *A* and *B*. The first child has a chance of one-half of getting *A* and one-half of getting *B*; so has the second child. It is like tossing two coins simultaneously. On the average, the result of every four throws is: two heads once; two tails once; and a head and a tail twice. So brothers and sisters, that is sibs, also share half their genes in common.

By extending this reasoning, we find that grandparent and grandchildren have one-quarter of their genes in common. So have uncle or aunt and nephew or niece. So have double first cousins.

Let us look at the important case of ordinary first cousins.

If one of the cousins, III 1, possesses a particular gene, the chance that she received it from her father, II 2, is 1/2. If he carries it, the chance that his sister, II 3, also carries it is 1/2. If she carries it, the chance that she transmitted it to her son III 2, is 1/2. $1/2 \times 1/2 \times 1/2 = 1/8$; hence, if a person bears a particular gene, the chance of encountering it in a cousin is 1/8. We can generalize this: first cousins have one-eighth of their genes in common.

As blood-relationship gets more remote, the proportion of genes in common falls off rapidly. Second cousins, that is, the offspring of two first cousins, have one-thirty-second of their genes in common; third cousins only one in one hundred and twenty-eight. The whole biological significance of consanguineous marriages lies in these figures and you can see how much more important are the close relationships and how relatively unimportant those which are more distant. There is another point, too; the essential thing is the degree of relationship of husband and wife. Previous consanguineous marriages in the lineage of either are irrelevant.

Human beings differ in their hereditary qualities because their genes differ and this applies both to normal variations and to abnormal characteristics. From the point of view of assessing the consequences of cousin marriage, however, it is the definitely abnormal genes which are important. Genes are stable things in the ordinary way, rather like atoms used to be in chemistry, but every now and then a physico-chemical change takes place and the gene changes its character. If this should happen in the germinal tract, the new gene, which has arisen by this process of mutation, is thereafter transmitted just like the old one. Confining our attention to abnormal genes we see that there are two possibilities. First, the organism may require the two normal unmutated genes for its normal development. If, as the result of a mutation in a parent, it receives the abnormal gene, together with the corresponding normal gene from the other parent, the damage is done and the individual is abnormal. The good gene cannot overcome the effect of the bad one. Should the effect of the bad gene be so serious that the abnormal individual cannot live or grow up, that is the end. It cannot be transmitted further. If, however, the effect is less serious we see the direct transmission from generation to generation of something like polydactyly or congenital cataract.

But the transmission of these dominant abnormalities is irrelevant to our subject. Effectively, it is inheritance from one parent only, and so it does not matter whether they are related or not. It is the other possibility, the recessive defect, which concerns us. It often happens that there is a sort of reserve of normality. Only one of the genes making up the pair is required for normal development. One gene can, in fact, do the work of two. The person who has one normal gene and one abnormal gene is outwardly perfectly normal. Only when two people happen to marry, both carrying the same abnormal gene, can the defect appear. Albinism is due to a recessive gene. The carriers, with one gene for albinism, are outwardly normal, but, of course, the gametes are of two kinds, produced in equal numbers. When two carriers happen to marry, these are the possibilities: first, the union of two gametes both with the normal gene, giving a perfectly normal child; second, a normal gamete, with an abnormal; or third, the other way round, abnormal with normal. Both these offspring are carriers. Finally both gametes may happen to carry the abnormal gene, and so we get the abnormal child, in this case the albino. The result, then, on the average, in terms of what we see, is the familiar Mendelian ratio of three to one—three normals to one affected. Thus, recessive defects are familial in their incidence. Father and mother are always, or nearly always, outwardly normal, but more than one child in the sibship is frequently affected.

Recessive defects are all rare, or very rare, for a reason I shall mention in a moment. There are, however, a great many of them and so they are not negligible in the aggregate. But though the defects appear rarely, because carriers do not often happen to marry, the genes themselves are not so very rare. This is because carriers are so much more numerous than the affected offspring themselves, that is, individuals with two abnormal genes. I think that it is easy to see this, without giving a formal proof. Actually, if one person in 10,000 is affected, one person in 50 in the population must carry the gene; if one person in 100,000 is affected, one person in 160 is a carrier; if one person in a million is affected, one person in 500 is a carrier. Now supposing a person is a carrier of one particular harmful recessive gene, with a frequency of carriers of one in 50, the chance that if he marries some unrelated person, she also will be a carrier is one in 50; but if he marries a first cousin, the chance is appreciably greater; actually, it is one in eight, as we saw a moment ago. In other words, whereas one marriage in 2,500 between unrelated persons is capable of yielding affected offspring, one marriage in 400 between first cousins is capable of doing so. If the abnormality is much rarer, say one in a million, one marriage in a quarter of a million between unrelated persons is capable of yielding affected offspring, compared to one marriage in 4,000 between first cousins.

It is indeed found that with rare recessive defects there is always an excess of consanguineous marriages between the parents as compared with the rate of cousin marriages in the population generally. If the defect is really very rare, say alkaptonuria, with an incidence of about one in a million, we expect and find that no less than about 40 per cent of all affected people are the offspring of first-cousin marriages.

So we see that an increased chance of the unexpected appearance of some recessive defect or other—and many of them are serious and unpleasant—is an inescapable consequence of the marriage of persons related by blood. What matters to individuals, however, is not by how much a small risk is relatively increased, but how great may be the total absolute risk. In fact, the increased absolute risk is not a very big one. The chance that any random pregnancy will end with some severe congenital abnormality or other, or that some serious developmental abnormality will manifest itself in early life, is about one in forty, and the added risk of a recessive defect turning up amongst the offspring of first cousins is probably less than this. Provided, of course, that there is no history of a recessive defect having already appeared in the family, the increase in the risk over and above the inevitable risks run by any couple who have a child is not of such an order that it need deter sensible and informed people and need not outweigh other considerations favourable to the marriage. We have a rather useful standard of comparison. In this country no fewer than one marriage in every eight is a marriage between a Rhesus negative woman and a Rhesus positive man. The consequent risk of haemolytic disease in second and later children is much greater than the risk of an undesirable recessive in a marriage of first cousins.

So we may conclude that the increased risk, though real, is relatively small. But though it may be ignored by individuals, even sensible and informed individuals, what about the consequences to the community? Let us think for a moment in an entirely theoretical sort of way whether it might be a good thing for the community if marriages of first cousins were prohibited. Of course, it is perfectly true that if marriages of first cousins were prohibited the total amount of recessive abnormality of all kinds in the population would be reduced to a certain extent. But we have to remember something else. Natural mutation is going on all the time, at the rates characteristic for each gene. Harmful genes are continuously being fed into the population, this being balanced by loss of genes, owing to the reduced fertility of their possessors. There is an equilibrium in these things. Incidentally, here is the reason why simply inherited defects are rare: it is because mutation is rare. The result of cutting down to some extent the proportion of affected children, those with two abnormal genes, would mean instead a corresponding increase in the number of carriers. Finally, after a long time, a new equilibrium would be reached. The original rate of manifestation would finally be restored; but there would now be many more carriers. A return to smaller populations and smaller breeding units would mean quite an outbreak of recessive defects of all kinds. So it does not seem that there is any strong case for interfering with the small amounts of inbreeding permitted and practised—that is important, practised rather than permitted—in our community.

This leads to a point which I might have mentioned before, but have postponed. How much consanguineous marriage is there in our population and is that amount changing? It certainly is. A hundred years ago, the proportion of first-cousin marriages was perhaps one per cent, 10 per 1,000. In a large survey carried out on hospital patients in the middle thirties the rate was 6 per 1,000. There is some evidence, though the numbers are small, that it has fallen further, even in the short time that has since elapsed, and may now be no more than 4 or 5 marriages in every 1,000. The reasons are fairly obvious. Growth in population, urbanization, and increased mobility with recent developments of transport, all mean that the average person meets and has the chance of marrying an ever-widening selection of members of the opposite sex, and so is less likely to choose someone related by blood. The amount of recessive abnormality of all kinds must, in fact, be less than it was last century.

Now you may well ask: "How does all this fit in with the experience of the animal breeder? Doesn't he practice inbreeding and line-breeding, often of the closest kind, and on a vast scale? Hasn't it been an essential and very important part of breed improvement? Is there no possible application, talking quite theoretically, to the human species?" All this is perfectly true. Inbreeding is essential if fixity of type is to be attained. The extent to which it has been carried out in domesticated animals is in fact amazing. If we were to take at random any two Shorthorn cattle from any part of the world, the resemblance between them, talking in averages, would be greater than the average resemblance between father and son in that breed 150 or 200 years ago. In some domestic breeds line-breeding has been practised to such an extent that more than a quarter of all the genes in the breed are derived from one individual, that is, an individual who is effectively the grandfather of the whole breed. There are even breeds in which more than half the genes come from one individual, that is, genetically speaking, he is the father of the entire breed. The animal breeder secures fixity of the type he desires in this way; but in the process many individuals have to be sacrificed, partly in exposing and eliminating undesirable recessives. But he does not mind. In many circumstances it is well worth sacrificing fifty failures in order to secure one outstanding success. This could hardly in any conceivable circumstances apply to man.

But there is something even more important. The breeder knows what he wants: beef from the ox, milk from the dairy cow, speed from the racehorse or greyhound, eggs from the hen. Usually he wants one essential thing from each. Even when he only wants two he often finds himself in considerable difficulty. The successful dual-purpose animal is notoriously difficult to achieve: dual-purpose cattle, for example, bred for meat and milk simultaneously; or dual-purpose sheep, bred for mutton and wool. In man we do not want one thing, or two things, but hundreds of things.

What man has done to his domesticated animals is in fact remarkable: the floods of milk poured out by the dairy cow, the hundreds of eggs laid by the hen, the obesity of the prize ox. Even worse, the coat of the sheep bred for wool, a coat which renders it susceptible to the attacks of the blowfly, whose larvae eat it alive; a coat that would entangle it in every thorn bush, and which, if not removed periodically by the hand of man, would quite literally strangle its unfortunate owner. Worst of all, perhaps, are the ornamental breeds, bred for what must have been regarded as quaint and appealing oddities, but which are in fact pathological monstrosities; the Bulldog or the Pekinese, for example. What would happen if there were a return to primitive conditions if the protection of man were removed? Surely all these freaks would join in oblivion the dinosaur, the mastodon and the sabre-toothed tiger. In contrast to this, perhaps the most valuable characteristic of our species is its variability, so that different people can specialize successfully in hundreds of different tasks. Then there is the other all-important aspect of variability—the ability of the species to adapt itself to changing conditions. Flexibility is essential to survival, and the only way to secure and retain it is outbreeding or something near it.

The biological implications of cousin marriage were considered in terms of harmful recessive genes; and indeed this is the most important thing from the practical point of view. There is no time left to consider hereditary variations within the range of normality, so I will just say this. There are, of course, all kinds of interesting theoretical points, and some not so theoretical. But for the individual couple contemplating a cousin marriage there is little or nothing to worry about except the harmful recessive genes. From the point of view of the community, there would indeed be problems, and of an interesting kind, if a very high proportion of the population married their cousins. But as we have seen, this is not so. Outbreeding is the rule and the relatively very small amounts of inbreeding actually practised do not have any more than a small effect on the distribution of normal, usually continuously distributed, characteristics, which are partly determined by heredity, and which distinguish one normal person from another.

I have come to the end of this brief review of cousin marriage and you may feel that I did not choose a very important subject; certainly not a controversial one. There is no burning problem here. But may there not be some kind of a lesson in that very fact? It is, after all, something that is related to very deep personal feelings. There has been trouble at times, but there is little or none now. We can consider biological implications in the calmest and most detached way. There is nothing here to produce that catch of the breath and rise of blood-pressure on opening the morning paper. Perhaps in some distant future—though there is little sign of it now—many other problems of life and conduct will fall into that happy state where there is an interesting past, but, to quote *1066 and All That*, " history has now stopped ".

## Figures and Tables

**Figure f1:**